# Experimental Infection of Common Eider Ducklings with Wellfleet Bay Virus, a Newly Characterized Orthomyxovirus

**DOI:** 10.3201/eid2312.160366

**Published:** 2017-12

**Authors:** Valerie Shearn-Bochsler, Hon Sang Ip, Anne Ballmann, Jeffrey S. Hall, Andrew B. Allison, Jennifer Ballard, Julie C. Ellis, Robert Cook, Samantha E.J. Gibbs, Chris Dwyer

**Affiliations:** US Geological Survey National Wildlife Health Center, Madison, Wisconsin, USA (V. Shearn-Bochsler, H.S. Ip, A. Ballmann, J.S. Hall);; Cornell University, Ithaca, New York, USA (A.B. Allison); Arkansas Game and Fish Commission, Little Rock, Arkansas, USA (J. Ballard);; Tufts University, North Grafton, Massachusetts, USA (J.C. Ellis);; National Park Service, Wellfleet, Massachusetts, USA (R. Cook);; US Fish and Wildlife Service, Fort Collins, Colorado, USA (S.E.J. Gibbs);; US Fish and Wildlife Service, Hadley, Massachusetts, USA (C. Dwyer)

**Keywords:** Common eider, hepatic necrosis, mass mortality, orthomyxovirus, *Quaranjavirus*, *Somateria mollissima*, ducklings, Wellfleet Bay virus, viruses

## Abstract

Wellfleet Bay virus (WFBV), a novel orthomyxovirus in the genus *Quaranjavirus*, was first isolated in 2006 from carcasses of common eider (*Somateria mollissima*) during a mortality event in Wellfleet Bay (Barnstable County, Massachusetts, USA) and has since been repeatedly isolated during recurrent mortality events in this location. Hepatic, pancreatic, splenic, and intestinal necrosis was observed in dead eiders. We inoculated 6-week-old common eider ducklings with WFBV in an attempt to recreate the naturally occurring disease. Approximately 25% of inoculated eiders had onset of clinical disease and required euthanasia; an additional 18.75% were adversely affected based on net weight loss during the trial. Control ducklings did not become infected and did not have clinical disease. Infected ducklings with clinical disease had pathologic lesions consistent with those observed during natural mortality events. WFBV was reisolated from 37.5% of the inoculated ducklings. Ducklings surviving to 5 days postinoculation developed serum antibody titers to WFBV.

The common eider (*Somateria mollissima*) is a large sea duck with a circumpolar distribution. Eiders nest in dense colonies, have generally low reproductive rates but high annual survival rates, and feed on filter-feeding bivalves, sea urchins, starfish, and crabs (*1*). The American race (subspecies *S. m. dresseri*) breeds on islands from the south central coast of Labrador, Canada, south to Massachusetts, USA, with greatest numbers breeding in the Gulf of St. Lawrence, St. Lawrence estuary, New Brunswick, and Nova Scotia (*2*). These birds typically overwinter from Newfoundland, Canada, south to New York, USA, with large rafts of >100,000 eiders congregating annually in the open waters off Cape Cod, Massachusetts (*1*).

The earliest record of death affecting a large number of eiders off Cape Cod occurred during September 1956–March 1957 (*3*). The next reports were not until the mid-1980s and early 1990s, when ≈100 birds (predominantly eiders) died in 2 separate events occurring in midwinter and late spring. Death in both events was primarily attributed to emaciation, although acanthocephaliasis (infestation with intestinal thorny-headed worms) was also present (*4*). Since 1998, however, die-offs of common eiders have been reported with increasing frequency on Cape Cod, such that they have become an almost annual (or biannual) occurrence since 2006. This increase in reports likely coincides with the initiation of active beach surveillance at several locations around the cape, which began after the isolation of a novel virus from eiders during the fall 2006 mass mortality event at Great Island in Wellfleet Bay, Massachusetts, USA (41.9048°N, 70.0687°W) (*5*). Most eiders were found dead, but a few live birds displayed nonspecific clinical signs, including incoordination, lethargy, and respiratory distress. Four common eider carcasses recovered from Great Island were submitted to the US Geological Survey’s National Wildlife Health Center (NWHC; Madison, WI, USA) for cause-of-death determination. Acute hepatic necrosis and variable pancreatic, splenic, and intestinal necrosis were diagnosed in all 4 of the submitted eiders, and a novel enveloped RNA virus, since named Wellfleet Bay virus (WFBV), was isolated from the liver of 2 of the 4 birds. Genetic analysis of WFBV demonstrated that it is most closely related to members of the *Quaranjavirus* genus within the family *Orthomyxoviridae* (*6*). Since 2006, the NWHC has diagnosed hepatic necrosis and variable splenic, intestinal, and pancreatic necrosis in common eiders from which WFBV was isolated during 4 separate mass mortality events occurring at Wellfleet Bay during September 2007–October 2011. We aimed to determine the pathogenicity of WFBV by experimentally inoculating common eider ducklings and observing clinical progression and pathologic changes after challenge.

## Materials and Methods

### Animals

We obtained the 22 common eiders used for this study by collecting eggs from wild breeding colonies in Maine, USA. We hatched and raised the ducklings to 4–5 weeks of age in a quarantined facility (Livingston Ripley Waterfowl Conservancy, Litchfield, CT, USA). We then transported the ducklings in an air-conditioned vehicle to the NWHC, where the infection trial was carried out. We acclimated the ducklings at the NWHC Biosafety Level 3 research facility for 7 days. We inoculated 16 ducklings with WFBV, housed 1 sham-inoculated duckling with the inoculated ducklings to act as a contact transmission control, and housed 5 sham-inoculated ducklings together in a separate room as controls. The NWHC–Animal Care and Use Committee approved animal use and protocols for this study (tracking no. EP100316).

We administered inoculations on day 0 of the infection trial. We randomly selected 6 inoculated ducklings (3 groups of 2) at the beginning of the study for euthanasia and necropsy on days 2, 4, and 7 postinoculation to determine pathologic changes and pathogenesis of the infection; we similarly chose 2 control ducklings and euthanized 1 on day 2 and 1 on day 4. We obtained health scores twice daily for all birds, and we weighed each eider daily. Health scores ranged 0–3, with 0 indicating normal, 1 indicating mild to moderate clinical abnormalities, 2 indicating severe clinical abnormalities, and 3 indicating death. We also euthanized any eiders not randomly scheduled for euthanasia before the end of the trial that reached a predetermined humane endpoint during the trial. We defined the humane endpoint as weight loss >30%, anorexia, lethargy, or other major illness lasting for at most 24 hours. We performed euthanasia by using CO_2_ inhalation followed by cervical dislocation. We concluded the trial at 10 days postinoculation (DPI), at which time we euthanized all remaining eiders.

### Virus

WFBV was first isolated from common eiders with hepatic necrosis in 2006, when it was isolated from liver homogenate inoculated onto Muscovy duck embryonic fibroblasts (*7*) and propagated in embryonating chicken eggs. The virus used for the inoculum in this trial was from a fourth-passage stock grown up in Muscovy cells originally derived from an eider carcass (NWHC case no. 22565, accession no. 002) collected in May 2009 during a mass mortality event near Wellfleet Bay. Because the natural route of infection (i.e., ingestion, inhalation, or vectorborne transmission) of WFBV is not known, we used an adaptation of the standard World Organisation for Animal Health (OIE) procedure for the determination of pathogenicity of avian influenza virus in poultry (*8*). We inoculated each of sixteen 6–7-week-old eider ducklings with WFBV by 3 routes simultaneously (intravenous, intratracheal, and oral). We administered a volume of 0.1 mL of inocula (3 × 10^6^ 50% egg infective dose/mL) by each route, for total virus amounts of 1 × 10^6^ 50% egg infective dose per bird. We inoculated the 5 control eiders housed separately from the exposed birds and the 1 contact transmission bird with a sham solution in a similar manner.

### Biologic Sample Collection and Analysis

We obtained daily oral and cloacal swabs before inoculation (0 DPI) through 10 DPI; placed them in a solution of viral isolation media (Eagle minimum essential medium, 10% fetal bovine serum, and 4% antibiotic–antimycotic solution, (all from Sigma-Aldrich, St. Louis, MO, USA); and froze them at −80°C. We also obtained 1 mL of venous blood from all eider ducklings before inoculation, at 5 DPI, and again at 10 DPI. We placed blood into serum tubes (Becton Dickinson, Franklin Lakes, NJ, USA) and separated the serum from the cellular fraction before storage at −30°C. We obtained oral and cloacal swabs daily and placed them into virus transport media. We collected 1 mL of venous blood from all eiders at 5 DPI and again at 10 DPI, collected the samples into serum tubes, and separated the serum. For eiders euthanized before 10 DPI, we collected serum before euthanasia. We performed antibody detection by using a modified β microneutralization assay (*9*). The antigen was a fourth-passage WFBV strain diluted to 50% tissue culture infective doses of 10^3^/25 µL in Eagle minimum essential medium. We serially diluted serum samples from 1:4 to 1:256 and tested them in quadruplicate. We used Vero M cells as the biologic indicator.

We performed postmortem examination on each eider after euthanasia, aseptically collecting cloacal and oropharyngeal swabs as well as tissue samples, including esophagus, intestine, liver, kidney, and spleen tissue from each bird and placing them into 1 mL of virus isolation media. We collected tissue samples (lungs, trachea, heart, brain, liver, kidneys, spleen, crop, proventriculus, gizzard, small and large intestine, pancreas, thymus, bursa of Fabricius, adrenal glands, and gonads) from each bird, fixed the samples in neutral-buffered 10% formalin solution, and processed them by standard methods for histopathologic examination. We examined slides by light microscopy and stained for WFBV any slides showing lesions by using immunohistochemistry as previously described (i.e., anti-WFBV polyclonal antibody mouse hyperimmune ascites fluid) (*6*).

## Results

### Weight Changes

All control eiders gained weight during the trial. Weight gains ranged from 105% to 130% (10-day average 118%). We euthanized 4 (25%) of the 16 inoculated eiders because of clinical deterioration during the 10-day trial period. These 4 eiders (band nos. 16, 22, 29, and 37) all lost weight steadily from the time of inoculation to the time of euthanasia; weight loss ranged from 7% to 20%. Of the remaining inoculated birds, 3 had net weight loss at the time of scheduled euthanasia. The remaining 9 virus-inoculated birds had all gained weight at the time of euthanasia (range 101%−137%, average 120%). Thus, of the 16 inoculated eider ducklings, 43.75% had a net decrease in weight during the trial. The single contact transmission control eider had a net gain in weight (16%) over the course of the trial.

### Disease

Control ducklings remained clinically healthy throughout the trial, whereas 25% of virus-inoculated eiders had onset of severe clinical signs and were euthanized. We began to observe clinical signs in several inoculated birds by 3 DPI. Two eider ducklings (band nos. 16 and 59) were lethargic, ataxic, and frequently had their eyes closed. At 4 DPI, we euthanized 1 of these eiders (band no. 16) and 1 additional eider (band no. 37) because of clinical deterioration (e.g., weak, not moving, and having pale mucous membranes); the second affected eider from 3 DPI (band no. 59) appeared to be recovering. At 5 DPI, an additional inoculated eider (band no. 22) developed an irregular gait; by 7 DPI, this bird was stumbling, reluctant to move, and had pale mucous membranes and was euthanized. Also at 7 DPI, another eider (band no. 29) became weak, pale, and reluctant to move and was euthanized. All remaining inoculated birds and the 1 contact transmission control were clinically normal throughout the trial.

### Virus Isolation

We detected shedding of WFBV by oropharyngeal swab (band no. 32) at 2 DPI and by cloacal swab (band no. 16) at 4 DPI. We isolated virus from multiple tissues (kidney, liver, spleen, esophagus, intestine) of 2 eiders (band nos. 27 and 52) that were euthanized at 2 DPI. After 2 DPI, virus isolation was confined to single tissues from 3 separate birds, at 4 DPI (band no. 16, esophagus), 7 DPI (band no. 48, kidney), and 10 DPI (band no. 34, intestine) (Table 1). We did not detect virus on swabs or tissues taken from control birds housed separately from inoculated birds or from the contact transmission control bird.

**Table 1:** Wellfleet Bay virus serum antibody titers and virus isolation results in common eider (*Somateria mollissima*) ducklings experimentally infected with Wellfleet Bay virus, by specimen or tissue type, day of trial, and band number of duckling*

Day of trial and band no.	Serum Ab titer	Specimen or tissue type						
		OP swab	CL swab	Esophagus	Intestine	Kidney	Liver	Spleen
Day 2								
27	Neg	0	0	0	2.00 × 10^3^	3.40 × 10^4^	2.00 × 10^4^	2.00 × 10^4^
32	NT	9.28 × 10^2^	0	NT	NT	NT	NT	NT
52	Neg	0	0	9.28 × 10^2^	6.32 × 10^2^	6.32 × 10^2^	6.23 × 10^2^	9.28 × 10^4^
Day 4								
16†	1:160	0	6.23 × 10	9.28 × 10^2^	0	0	0	0
25	Neg	0	0	0	0	0	0	0
37†	1:320	0	0	0	0	0	0	0
42	1:160	0	0	0	0	0	0	0
Day 5								
22	1:160	0	0	NT	NT	NT	NT	NT
29	1:160	0	0	NT	NT	NT	NT	NT
30	1:320	0	0	NT	NT	NT	NT	NT
32	1:160	0	0	NT	NT	NT	NT	NT
34	1:160	0	0	NT	NT	NT	NT	NT
43	1:320	0	0	NT	NT	NT	NT	NT
48	1:320	0	0	NT	NT	NT	NT	NT
50	1:320	0	0	NT	NT	NT	NT	NT
59	1:160	0	0	NT	NT	NT	NT	NT
60	>1:20, <1:160	0	0	NT	NT	NT	NT	NT
11‡	Neg	0	0	NT	NT	NT	NT	NT
Day 7								
22†	1:160	0	0	0	0	0	0	0
29†	1:160	0	0	0	0	0	0	0
48	1:320	0	0	0	0	4.31 × 10^2^	0	0
Day 10								
30	>1:640	0	0	0	0	0	0	0
32	1:160	0	0	0	0	0	0	0
34	>1:640	0	0	0	6.32 × 10^3^	0	0	0
43	1:80	0	0	0	0	0	0	0
50	1:160	0	0	0	0	0	0	0
59	>1:640	0	0	0	0	0	0	0
60	>1:640	0	0	0	0	0	0	0
11‡	Neg	0	0	0	0	0	0	0

*Virus isolate results are expressed as PFU/mL. Negative serum Ab titer result defined as titer <1:5. Ab, antibody; CL, cloacal; NT, not tested; Neg, negative; OP, oropharyngeal. †Euthanized because of illness. ‡Contact transmission control.

### Serum Antibody Detection

All virus-inoculated eiders developed serum antibodies to WFBV by 5 DPI. Antibodies persisted and remained stable or increased in all but 2 of the eiders that survived to the end of the trial (Table 1). Separately housed control eiders and the single contact transmission control eider did not develop detectable antibodies to WFBV.

### Pathologic Changes

**Figure 1:**
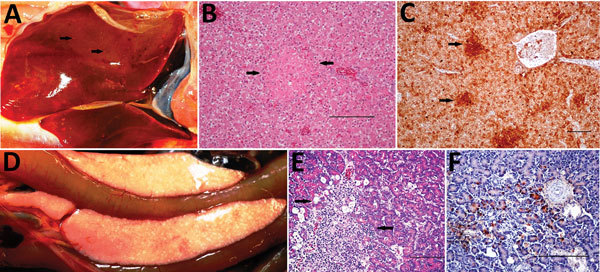
Gross, histopathologic, and immunohistochemical (IHC) findings in common eider (*Somateria mollissima*) ducklings experimentally infected with Wellfleet Bay virus (2 days postinoculation). A) Liver, enlarged, showing multifocal pinpoint areas of necrosis (arrows). B) Hematoxylin and eosin stain of liver tissue, showing focal hepatocellular necrosis (arrows). C) IHC stain of liver tissue, showing positive immunolabeling for Wellfleet Bay virus with multifocal staining of hepatocytes (arrows). D) Pancreas showing multifocal to coalescing acute necrosis. E) Hematoxylin and eosin stain of pancreas tissue, showing focally extensive necrosis of exocrine cells (arrows). F) IHC stain of pancreas tissue, showing positive immunolabeling for Wellfleet Bay virus in exocrine cells. Scale bars in panels B, C, E, and F indicate 100 μm.

**Figure 2:**
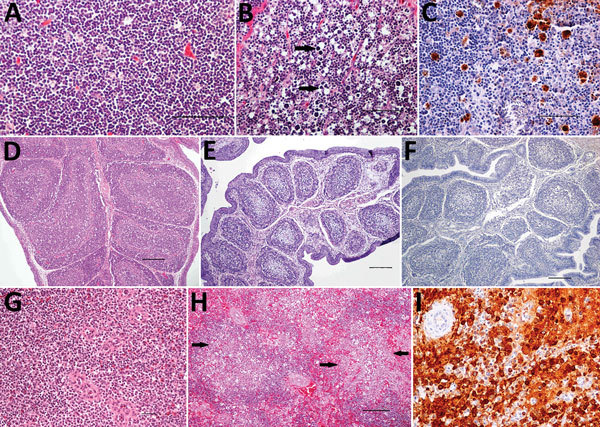
Histopathologic and immunohistochemical (IHC) findings in lymphoid organs of control and infected common eider (*Somateria mollissima*) ducklings experimentally infected with Wellfleet Bay virus (WFBV) (2 days postinoculation). A) Hematoxylin and eosin (H&E) stain of thymic cortex tissue from a control duckling. B) H&E stain of thymic cortex tissue from an infected duckling, showing marked multifocal acute apoptosis of lymphocytes (arrows). C) IHC stain of thymus tissue from an infected duckling, showing positive immunolabeling for WFBV in thymocytes. D) H&E stain of bursa of Fabricius tissue from a control duckling. E) H&E stain of bursa of Fabricius tissue from an infected duckling, showing marked diffuse lymphoid depletion. F) IHC stain of bursa of Fabricius tissue from an infected duckling showing negative immunolabeling for WFBV. G) H&E stain of spleen tissue from a control duckling. H) H&E stain of spleen tissue from an infected duckling, showing multifocal to coalescing acute necrosis (arrows). I) IHC stain of spleen tissue, showing positive immunolabeling for WFBV. I, original magnification ×200. Scale bars in panels A, B, and C indicate 50 µm and in panels D, E, F, G, and H indicate 100 µm.

We observed severe gross and histopathologic abnormalities in both of the eiders euthanized at 2 DPI, before clinical signs were observed in any of the virus-inoculated eiders. This finding suggests that viremia (we isolated virus from multiple tissues in both of these birds) and tissue damage occur very early in the course of infection. Pathologic abnormalities in these 2 eiders and in eiders showing signs of clinical illness later in the trial were consistent with those observed in adult common eiders from naturally occurring mass mortality events, including multifocal hepatic necrosis that progressed to hepatitis and pancreatic necrosis that progressed to pancreatitis. Immunohistochemical staining confirmed the presence of virus associated with these lesions (Figure 1). We scored the severity of hepatic lesions by histopathologic assessment of the number and extent of necrotic foci within sections of liver, with marked hepatic necrosis/hepatitis defined as affecting >30% of the hepatic parenchyma, moderate necrosis/hepatitis defined as affecting 20%–30%, and mild necrosis/hepatitis defined as affecting >0% to <20% (Table 2). Acute, marked thymic lymphocytic apoptosis, splenic necrosis, and lymphoid depletion in the bursa of Fabricius were also present in both birds; immunohistochemical staining for WFBV was positive in the thymus and spleen but not in the bursa of Fabricius. We observed no abnormalities in the control bird euthanized at 2 DPI, and both the thymus and bursa of Fabricius of this bird were robustly populated with lymphocytes (Figure 2).

**Table 2:** Necropsy findings in common eider (*Somateria mollissima*) ducklings experimentally infected with Wellfleet Bay virus and control ducklings, by control or inoculated status and band number of duckling*

Status and band no.	DPI	Weight change, %†	Health score‡	Virus isolated	Liver necrosis	Lymphoid depletion	Aspergillosis	Malaria	Muscle parasite
Control									
23	2	+4.76	0	No	–	–	+	–	–
36	4	+22.06	0	No	–	–	+	++	++
21	10	+20.63	0	No	–	–	+	++	++
28	10	+19.29	0	No	–	–	–	–	–
33	10	+14.02	0	No	–	–	–	++	+
Inoculated									
27	2	+2.88	0	Yes	+++	+++	++	–	–
52	2	−5.08	0	Yes	+++	+++	+	–	–
16§	4	−6.52	2	Yes	++	+++	+	+++	++
25	4	−13.60	0	No	++	+++	–	+	++
37§	4	−13.97	2	No	++	+++	–	–	++
42	4	−14.02	0	No	++	+++	+	–	–
22§	7	−20.14	2	No	++	+++	–	++	+++
29§	7	−17.87	2	No	++	+++	–	–	+
48	7	+1.03	0	Yes	–	++	+	++	+++
30	10	+13.25	0	No	++	+	+++	–	–
32	10	+17.20	0	No	–	+	++	–	–
34	10	+36.97	0	Yes	+	+	+	+	++
43	10	+17.45	0	No	+	+	+	+	+++
50	10	+29.27	0	No	–	–	++	++	+
59	10	+27.13	0	No	–	–	++	++	+
60	10	+0.55	0	No	–	+	+++	–	–
Contact transmission control									
11	10	+15.66	0	N	–	–	+++	++	+

*DPI, days postinoculation; +, mild; ++, moderate; +++, marked; –, negative. †From day 0 to date of euthanasia. ‡Health scores ranged from 0–3, with 0 indicating normal, 1 indicating mild to moderate clinical abnormalities, 2 indicating severe clinical abnormalities, and 3 indicating death. §Euthanized because of illness.

Lymphoid depletion was a major finding in virus-inoculated eiders but was not present in the controls; all 5 control eiders had robust lymphoid organs at the time of scheduled euthanasia (at 2, 4, and 10 DPI). In contrast, inoculated birds that were euthanized at 2 DPI (2 birds) or 4 DPI (4 birds) and 2 of 3 birds euthanized at 7 DPI had marked lymphoid depletion in spleen, thymus, and bursa of Fabricius. Immunohistochemical stains confirmed the presence of WFBV in thymus and spleen but not in the bursa of Fabricius (Figure 2) The severity of lymphoid depletion was scored as marked if spleen, thymus, and bursa of Fabricius all showed an estimated >75% decrease in expected numbers of lymphocytes, moderate if severity of depletion varied between lymphoid organs but was estimated as a >50% decrease in lymphocytes, and mild if some lymphoid organs showed very little depletion whereas others showed moderate depletion (Table 2). The 2 birds euthanized at 7 DPI that had marked lymphoid depletion showed clinical signs, including at the time of euthanasia; the third bird had only mild to moderate lymphoid depletion and was clinically normal. By 10 DPI, all remaining virus-inoculated birds were clinically normal and had no or only mild lymphoid depletion.

### Co-infections

The presence of 3 unanticipated pathogens in virus-inoculated, control, and contact transmission control eiders complicated the interpretation of pathologic findings in some of the eiders euthanized after 2 DPI. Avian malaria (caused by infestation with *Plasmodium* sp. protozoa), aspergillosis (infection with *Aspergillus fumigatus*), and an unidentified skeletal muscle protozoan were variably present in control and inoculated ducklings. The skeletal muscle protozoan was present as degenerating, often calcified cysts invaded by macrophages and lymphocytes. We rated the severity of protozoal infection in the following manner: we removed a 2 × 1 × 1 cm section of muscle from the cranioventral flexor carpi ulnaris muscle, where the parasites were most commonly observed on gross examination, and placed it in formalin. We performed histopathologic examinations on 3 cross-sections of this tissue from each bird and counted the number of discrete protozoal cysts present. Marked infestations were diagnosed as >50 total cysts or mineralized cyst remains with associated granulomatous inflammation, moderate as 20–50, and mild as 1–20. The contact transmission control eider and 4 of the 5 separately housed control ducklings had >1 co-infection, but all gained weight and remained clinically healthy throughout the trial. Two of the 4 virus-inoculated eiders that had clinical signs of illness had 1 co-infection of similar severity to those observed in controls; the other 2 had multiple co-infections. Co-infections in WFBV-inoculated eiders were more severe overall than those in control eiders on the basis of numbers of parasites present (avian malaria and muscle protozoa) as well as severity and extent of associated tissue damage (Table 2). Pathologic abnormalities typical of natural WFBV disease were also present in eiders with major co-infections, and immunohistochemical staining confirmed the presence of virus associated with these lesions.

## Discussion

This study determined the pathogenicity of WFBV, a novel orthomyxovirus in the genus *Quaranjavirus*, in common eiders by observing clinical progression and pathologic changes in experimentally inoculated eider ducklings over a 10-day period. We were able to determine that WFBV causes a unique, characteristic, and reproducible disease in this host.

Virus shedding occurred in only 2 infected eider ducklings very early in the course of infection with WFBV. This finding has practical implications; the diagnosis might easily be missed in naturally occurring infections if testing is confined to virus detection from oropharyngeal or cloacal swabs of dead or sick birds. Ducklings in this study did, however, develop serum antibody titers to WFBV by 5 DPI, and titers persisted through 10 DPI, when the experiment ended. Serologic testing would therefore be a useful tool for diagnosis of WFBV infection during a mass mortality event and for field surveillance.

Results of this study suggest that immune suppression might play an important role in the pathogenesis of WFBV disease in eiders. All eider ducklings that had onset of clinical disease had histopathologic evidence of marked lymphoid depletion in the spleen, thymus, and bursa of Fabricius. In contrast, inoculated ducklings that remained clinically healthy until 7 DPI or 10 DPI had no or only mild lymphoid depletion. None of the control eiders (those housed separately plus the contact transmission control) demonstrated lymphoid depletion despite concurrent parasitic or fungal infections. Immune suppression induced by WFBV infection might lead to a greater risk for disease under conditions of increased stress or through the exacerbation of preexisting conditions. Co-infections in eiders with disease tended to be more severe than in eiders that remained asymptomatic during the trial (Table 2), although 1 eider (band no. 29) had only a mild muscle protozoan infestation. We hypothesize that under natural conditions, eiders affected by some combination of malnutrition, migration, concurrent disease, or other stressors might develop disease when infected with WFBV at a higher rate than birds without additional stressors.

In 2010 in Australia, an orthomyxovirus with genetic and antigenic properties similar to WFBV (designated as Cygnet River virus [CyRV]) was isolated during an outbreak of salmonellosis (*Salmonella enterica* serovar Typhimurium) in Muscovy ducks (*Cairina moschata*) on Kangaroo Island, South Australia (*10*). Genetic and antigenic comparisons between WFBV and CyRV indicate that they might be genetic variants of the same virus (*6*). It has not yet been determined what, if any, role CyRV played in the Muscovy duck deaths, given that salmonellosis alone is capable of causing a mortality event in waterfowl (*11*); however, we suggest that the high death rate observed in ducks with salmonellosis (97%) indicates that co-infection with CyRV might have contributed to the severity of this mortality event.

WFBV is the first *Quaranjavirus* to be definitively shown to cause major disease in its host species. In addition to CyRV, other members of the genus include Quaranfil, Johnston Atoll, and Lake Chad viruses, all of which have been associated with colonial nesting avian species and their associated ticks (*12*). Quaranfil virus is particularly interesting from a public health standpoint. This virus was named for the village in Egypt from which it was first isolated in *Argas arboreus* ticks associated with a nesting colony of egrets (*Bubulcus ibis ibis*). Quaranfil virus was subsequently isolated from the blood of 2 young children with mild febrile illnesses (*13*). The children recovered without incident. Although these findings do not prove that illness in the children was caused by the Quaranfil virus, they do indicate that at least 1 virus in the *Quaranjavirus* genus can infect humans. This fact highlights the importance of surveillance for and diagnosis of emerging diseases in wildlife as a means of detecting new pathogens with the potential to cause disease in humans and in nonhuman species.

The potential importance of WFBV to common eider populations is not yet clear. A recent article reported that another cyclically occurring infectious disease, avian cholera (caused by the bacterium *Pasteurella multocida*), is likely to have a substantial negative effect on the breeding population of common eiders on Southampton Island, Nunavut, Canada (*14*). This observation might also prove to be true of WFBV; however, much remains to be determined regarding the prevalence and sublethal effects of this virus during the breeding season. 

Based on the results of our study, WFBV infection seems most likely to cause illness and death in eiders that are already physiologically stressed by migration or other natural phenomena or that contract what would otherwise be nonlethal co-infections. Further monitoring and surveillance of disease outbreaks associated with WFBV and research into its mode of transmission will increase our understanding of this virus and its potential for population-level effects.
